# Unusual Micronutrient Deficiencies as Causes of Anemia

**DOI:** 10.3390/nu18040664

**Published:** 2026-02-18

**Authors:** Ananya Datta Mitra, Ralph Green

**Affiliations:** Department of Pathology and Laboratory Medicine, University of California Davis School of Medicine, Sacramento, CA 95817, USA

**Keywords:** nutritional anemia, micronutrients

## Abstract

Anemia due to micronutrient deficiencies has received little attention in public health and modern-day clinical practice. Increasing numbers of persons, mainly in low- and middle-income nations, are faced with malnutrition and malabsorption syndromes, giving rise to various micronutrient deficiencies that can lead to anemias refractory to treatment with iron, folate or vitamin B12. Though relatively uncommon, such underlying nutrient deficiencies may be difficult to recognize as they can coexist with more common causes and there may be few or no specific clinical characteristics pinpointing a particular micronutrient. The main examples of these micronutrients contributing to a small but important burden of anemia are other B-group vitamins, pyridoxine and thiamine, ascorbic acid, the fat-soluble vitamins, A and E and other trace elements such as copper, zinc and selenium. The following review highlights the current state of knowledge and the relevance of these micronutrient deficiencies in the broader context of nutritional anemia.

## 1. Introduction

Although far less common than the major micronutrient deficiencies (iron, vitamin B12 and folate), deficiencies of other trace minerals and vitamin micronutrients play an important role in causing anemia. Red cell production to balance destruction maintains adequate red cell numbers to prevent anemia. This balance is maintained by the erythroid precursors in the bone marrow, which require adequate nutritional supplies. While the most critical nutritional support is provided by iron, vitamin B12 and folate, deficiencies of other nutrients, although less common, can lead to or exacerbate anemia. These include deficiencies of riboflavin, pyridoxine, thiamine, ascorbic acid, vitamin A and E, trace elements like copper, zinc and selenium and protein-energy malnutrition [1]. Such micronutrient deficiencies can also exist simultaneously, and it may be difficult to identify which deficiency is responsible for the anemia. Deficiencies of specific micronutrients are associated with tissue effects beyond anemia, and the existence of such manifestations may be helpful in identifying the nature of the underlying deficiency. Full consideration of these features lies beyond the scope of this review. Here, we describe some of the more prominent manifestations of uncommon micronutrient deficiencies as they pertain to anemia.

## 2. Anemia Caused by Other Vitamin Deficiencies

### 2.1. Vitamin A

Vitamin A deficiency is a public health problem in infants, children and reproductive-age women in developing countries [2,3]. In this demographic setting, iron deficiency can also coexist [4]. However, there is no known causal association between these two nutrients outside of being part of generalized malnutrition. In the United States, vitamin A deficiency can occur; however, its association with anemia is not well established.

Long-lasting deficiency of vitamin A can result in microcytic and hypochromic anemia with anisocytosis (changes in cell size) and poikilocytosis (changes in cell shape) and low serum iron like that in iron deficiency [5,6,7,8]. However, in vitamin A deficiency not associated with concomitant iron deficiency, the liver and bone marrow iron stores are increased with normal to low serum transferrin. Also, the anemia does not correct with exogenous iron therapy. Studies suggest that iron absorption can be hindered by vitamin A deficiency due to an alteration of gene expression responsible for intestinal iron absorption [9,10]. However, whether vitamin A truly enhances iron absorption is still a matter of debate [11,12,13]. It has been noted that the management of anemia with both vitamin A and iron creates a better response than the administration of either nutrient alone [14]. In keeping with its known role in promoting cellular differentiation, vitamin A has also been shown to promote the growth and differentiation of erythroid precursors [15].

### 2.2. Vitamin B (Excluding Vitamin B12 and Folate)

Although there is evidence of hematologic abnormalities in experimentally induced isolated deficiencies of other members of the vitamin B group in animals, there is no solid evidence linking individual nutritional deficiencies of pyridoxine, riboflavin, pantothenic acid, and niacin to anemia in humans.

### 2.3. Vitamin B6 Deficiency

Pyridoxal phosphate (vitamin B6) is a cofactor in numerous enzymatic reactions, including aminolevulinic acid synthesis in the heme pathway. Deficiency may result from inborn errors of metabolism, malabsorption (e.g., celiac disease, pancreaticoduodenectomy), renal dialysis or nephrosis with proteinuria (loss of albumin-bound vitamers), poor nutrition (including exclusive goat’s milk feeding), pregnancy, and certain medications such as isoniazid. Vitamin B6 deficiency can occur in infants, resulting in microcytotic hypochromic anemia [16], in pregnant women, and in malnourished patients, where it is associated with an anemia unresponsive to iron supplementation [17,18]. Such patients respond well to subsequent administration of vitamin B6.

Isoniazid-induced vitamin B6 deficiency and peripheral neuropathy can be corrected with large doses of pyridoxine [19]. The Centers for Disease Control and Prevention, (CDC) recommends the prescription of pyridoxine along with isoniazid. It has been reported that some patients with primary sideroblastic anemias also respond to the administration of pyridoxal phosphate, with a rapid reticulocyte response and sustained control of anemia [20].

Patients undergoing pancreaticoduodenectomy may have vitamin B6 deficiency due to a lack of absorption from the duodenum and proximal jejunum, which is responsive to the administration of pyridoxine [21]. Goat’s milk also can cause pyridoxine deficiency in conjunction with folate deficiency and has been reported in an infant fed exclusively with goat’s milk [22]. Other important causes of pyridoxine deficiency as noted above are renal dialysis and nephrosis with proteinuria, which cause a loss of albumin-bound vitamin B6 vitamers from the circulation [23].

### 2.4. Riboflavin

Riboflavin is unique among the water-soluble vitamins regarding its role in numerous redox reactions. Riboflavin deficiency results in a decrease in red cell glutathione reductase activity; however, this does not cause hemolytic anemia due to oxidant-mediated damage. Studies have shown that human subjects who were maintained on a riboflavin-deficient diet and fed on galactoflavin, a riboflavin antagonist, developed vacuolated erythroid progenitors followed by the development of pure red cell aplasia [24]. Riboflavin administration resulted in a reversal of this anemia. Riboflavin deficiency may cause a reduced utilization of iron from intracellular stores causing anemia [24,25,26].

Erythrocyte glutathione reductase activity (EGRac) in the fasting blood of healthy non-pregnant, non-breastfeeding women in Canada and Malaysia showed an inverse relation between EGRac and hemoglobin levels, and the authors concluded that the deficient biomarker status of riboflavin can predict which women are likely to present with anemia [27]. Reports have also suggested that riboflavin deficiency can interfere with the metabolism of other B vitamins like folate and vitamin B6 [28].

### 2.5. Pantothenic Acid Deficiency

Anemia does occur in rats with diets deficient in pantothenic acid [29]; however, pantothenic acid deficiency, when artificially induced in humans, is not associated with any anemia [30].

### 2.6. Niacin Deficiency

Niacin deficiency leads to pellagra, which is characterized by the “4D” triad of dermatitis, diarrhea, dementia and death. It is often seen in alcoholics, homeless persons, and patients suffering from malabsorption and can be a health problem in malnourished children. Occasionally pellagra is associated with anemia. Although this anemia is responsive to niacin therapy [31], it is, however, not clear whether the anemia is directly related to niacin deficiency or related to generalized malnutrition.

### 2.7. Thiamine Deficiency

Thiamine-responsive megaloblastic anemia syndrome (TRMA) or Rogers syndrome consists of a constellation of megaloblastic anemia, progressive sensorineural hearing loss, and diabetes mellitus and has been reported mostly in patients of Middle and Far Eastern origin. The megaloblastic anemia typically occurs in infants or adolescents and is characterized by severe anemia with thrombocytopenia with or without ring sideroblasts in the bone marrow [32]. The anemia generally responds to thiamine therapy; however, the macrocytosis is persistent, and a cessation of treatment leads to a recurrence of anemia. The pathogenesis of this condition appears to be due to a defect in the high-affinity thiamine transporter, affecting the synthesis of the ribose portion of nucleic acids, which then leads to cell-cycle arrest or apoptosis in marrow cells [33]. Anemia in these patients responds to a lifelong administration of oral thiamine (25–100 mg/day). All reported cases of thiamine-responsive megaloblastic anemia are linked to the SLC19A2 gene on chromosome 1q23.3, encoding the high-affinity thiamine transporter [34]. Interestingly, the reduced folate carrier (SLC19A1) and the thiamine transporters-1 and -2 (SLC19A2, SLC19A3) are postulated to have developed from the same family of solute carriers [35].

### 2.8. Vitamin C (Ascorbate)

Ascorbate or ascorbic acid is critical for maintaining the structural integrity of tissues in humans, who, like several other species including non-human primates, guinea pigs and fruit bats, cannot synthesize this nutrient and rely on exogenous sources for maintaining tissue integrity. Within 1–3 months of the onset of vitamin C deficiency, patients start showing symptoms of scurvy. Studies show that the majority of the patients with scurvy usually develops normocytic normochromic or macrocytic anemia [36], but this anemia is not reproducible in human subjects exposed to diets restricted in ascorbate alone [37]. The anemia is more related to a deficiency of folate [36]. Scorbutic patients with coexisting megaloblastic anemia were refractory to vitamin C therapy when they were maintained on a folate deficient diet, but showed a prompt hematological response when given 50 mcg/day folate [38].

Ascorbic acid maintains dihydrofolate reductase in its reduced or active form, which participates in the formation of tetrahydrofolate, the metabolically active form of folic acid. Patients with coexisting scurvy and megaloblastic anemia excrete 10-formylfolate as the key urinary folate metabolite. However, after ascorbic acid therapy, the main urinary folate metabolite in these patients is 5-methyltetrahydrofolate. Based on this observation, it was proposed that ascorbic acid prevents the irreversible oxidation of methyltetrahydrofolate to formylfolate [39]. Thus, ascorbate is essential not only to synthesize tetrahydrofolate but also to protect it from oxidation and thus to prevent megaloblastic anemia. Also, ascorbic acid only produces a hematologic response if adequate folate is present to interact with the ascorbic acid [40].

Furthermore, ascorbate plays an important role in non-heme iron absorption, and dietary ascorbate deficiency can lead to iron deficiency and anemia. Ascorbic acid potentiates iron absorption in the intestine by keeping iron in the more soluble reduced ferrous (Fe^2+^) state. Microcytic hypochromic anemia occurring in scurvy may require simultaneous therapy with iron and vitamin C to correct the anemia [41].

Moreover, ascorbic acid modulates transferrin-dependent iron uptake by mobilizing iron from endosomes, which delivers nearly all iron for erythropoiesis. Thus, a deficiency of ascorbate can lead to iron deficiency anemia [42]. Hemorrhage caused by a loss of capillary integrity in scurvy may further contribute to iron deficiency. Furthermore, in patients with the iron-refractory iron-deficiency anemia (IRIDA) phenotype caused by genetic defects in the TMPRSS6 gene, an improvement in the hematologic response to oral iron and vitamin C combination therapy in children has been described [43].

Patients receiving chronic blood transfusions and patients with high iron-containing diets have iron overload, which leads to a decrease in the levels of vitamin C in leukocytes due to rapid oxidation of ascorbate to oxalate [44]. Also noted is that ascorbate deficiency decreases the efficacy of the iron-chelating agent deferoxamine (desferrioxamine)-induced iron excretion and which is normalized with vitamin C supplementation [45,46]. Studies have shown that scurvy in patients with iron overload may protect them from tissue damage [47]. Iron accumulates in the monocyte–macrophage system in guinea pigs and Southern African subjects with nutritional vitamin C deficiency and dietary hemosiderosis and prevents injury to the parenchymal cells of the liver [48,49]. Thus, ascorbic acid should only be given following the initiation of an infusion of deferoxamine mesylate (Desferal) in patients with iron overload to avoid possible tissue damage caused by free radical generation resulting from sudden iron release. Whether this applies to the currently used oral iron chelators is not known. In the setting of prolonged inflammation, as occurs in many chronic infectious, autoimmune, or malignant conditions, iron homeostasis is altered through hepcidin-mediated pathways. Inflammatory cytokines, particularly interleukin-6, stimulate hepatic hepcidin production, which in turn degrades the iron exporter ferroportin on enterocytes and macrophages. This results in reduced intestinal iron absorption and sequestration of iron within reticuloendothelial cells, limiting its availability for erythropoiesis despite adequate or increased total body iron stores, a process central to the pathogenesis of anemia of inflammation [50,51]. Although not a true deficiency in total body iron, this functional iron deficiency state is clinically important because it may mimic iron deficiency anemia biochemically and requires a distinct therapeutic approach. Furthermore, studies suggest a mechanistic link between vitamin D and regulation of the hepcidin–ferroportin axis during chronic inflammation [50,52]. Proposed mechanisms include a direct suppression of hepcidin transcription and indirect effects via the downregulation of pro-inflammatory cytokines that stimulate hepcidin production [52,53]. Although early human studies demonstrate reductions in hepcidin following vitamin D supplementation, the downstream effects on iron indices and hemoglobin require confirmation in larger, longer-term clinical trials.

### 2.9. Vitamin E

Vitamin E, α-tocopherol, is a lipid-soluble constituent of cellular antioxidant systems in humans. It is present in lipid-rich diets and is not a recognized cofactor in any essential specific physiological reactions. Due to its fat-soluble nature, it is generally stored within the fatty tissues, and its deficiency in humans is very rare because of a rich dietary abundance of α-tocopherol in food, especially in vegetable oils. There is no recommended daily requirement of vitamin E, and intake varies from 5 to 7 mg depending on the polyunsaturated fatty acid content in the diet and the content of peroxidizable lipids in tissues [54,55]. The World Health Organization (WHO/FAO, 2004) considered that the data were not sufficient to set Population Reference Intakes (PRIs) for ‘vitamin E’, and proposed ‘best estimates of requirements’ of 10 and 7.5 mg α-TE/day for men and women, respectively [56]. Deficiency of this vitamin is relatively rare in humans and happens almost solely in people with an inherited or acquired condition that hampers their ability to absorb the vitamin (like in cystic fibrosis, short bowel syndrome or bile duct obstruction) and in those who have an impairment of dietary fat absorption or have disorders of fat metabolism.

Studies have shown that vitamin E deficiency can lead to hemolytic anemia in low-birth-weight infants fed with diets rich in polyunsaturated fatty acids. This usually manifests by 4–6 weeks of age and occurs mostly if iron supplements are being administered [57]. Vitamin E has been implicated in maintaining red blood cell membrane (RBC) integrity by preventing the oxidation of polyunsaturated fatty acids in the red cell membrane and thereby preventing premature erythrocyte lysis. Thus, vitamin E deficiency leads to fragmentation and other morphologic alterations of the erythrocytes [58]. There is also an associated thrombocytosis, and dorsal edema of the feet and pretibial area [59]. Reportedly, treatment with vitamin E results in a rapid rise in hemoglobin levels, decreased reticulocytosis as seen commonly in these infants, stabilization of the red cell lifespan, and correction of thrombocytosis and dorsal edema. It has been reported that vitamin E fortification of infant formulas has virtually eliminated vitamin E deficiency in preterm infants [60]; however, recently, a case of hemolytic anemia was reported in a preterm infant that responded to vitamin E supplementation [61].

Vitamin E deficiency is common in patients with cystic fibrosis due to chronic steatorrhea and pancreatic insufficiency, leading to severe anemia [62]. A moderate but statistically significant drop in red cell lifespan is observed in these patients. On average, the ^51^Cr half-life was reduced to 19 days (normal, ~30 days), which increased significantly to 27.5 days after vitamin E therapy [63]. A Cochrane review of four studies with a total of 141 participants highlights that vitamin E supplementation may lead to an increase in vitamin E levels in cystic fibrosis, which can prevent the serious complications of vitamin E deficiency, such as hemolytic anemia and cerebellar ataxia [64].

Vitamin E has been implicated in the prevention of red cell damage to oxidant stress in hereditary hemolytic anemias, leading to oxidant damage, and it has been reported that chronic administration of vitamin E 400–800 U/day was associated with an improvement of the red cell lifespan in some [65,66], but not all [67], studies of patients with hereditary hemolytic anemias related to glutathione synthetase deficiency or glucose-6-phosphate dehydrogenase deficiency. Notably, in patients with sickle cell anemia, daily administration of vitamin E (450 U/day for 6–36 weeks) resulted in a profound reduction in the number of circulating irreversibly sickled erythrocytes [68]. Studies have also noted that serum tocopherol is lower in adults with sickle cell anemia compared with normal counterparts [69,70]. Moreover, irreversibly sickled cells were notably more numerous in children with sickle cell anemia and concomitant vitamin E deficiency than in children without vitamin E deficiency [71].

## 3. Anemia Caused by Trace Metal Deficiencies

### 3.1. Copper

Copper is present in several metalloproteins and is associated with numerous copper-containing enzymes responsible for important biological pathways, including neurotransmission, generation of energy and development of the extracellular matrix. Copper-containing enzymes include cytochrome C oxidase, dopamine β-hydroxylase, urate oxidase, tyrosine and lysyl oxidase, ascorbic acid oxidase, and superoxide dismutase. Most of the copper in circulation is carried bound to ceruloplasmin (also known as ferroxidase I (FeOxI)), an α2-globulin. Copper transporters (CTR) in the SLC31 family play a vital role in copper transport, compartmentalization and utilization [72].

Copper is also essential for iron absorption and utilization. The ceruloplasmin homologue hephaestin converts iron to the ferric (Fe^3+^) state, which is required for its transport by transferrin, and thereby participates in the absorption, uptake and utilization of iron [73].

Studies have shown that malnourished children [74], patients with total parenteral nutrition [75,76,77] and patients post gastrectomy or bariatric surgeries can develop copper deficiency [78,79]. Lack of copper can lead to iron-refractory anemia with hypoferremia, neutropenia and osteoporosis [76]. This anemia is typically normocytic or macrocytic and rarely microcytic, with varying severities depending on body copper levels, and the bone marrow is hypercellular, with the presence of ring sideroblasts and vacuolated erythroid and granulocytic precursors [75,78,80]. The effect of copper deficiency on granulocytic (white cell) production characteristically shows left-shifted myelopoiesis, resulting in myeloid arrest and neutropenia, which results from decreased survival of mature polymorphonuclear cells and inhibition of differentiation and self-renewal of hematopoietic progenitor cells [81,82].

Also notable in copper deficiency are hemosiderin-laden plasma cells in the marrow, and hypogranular and hypolobated neutrophils with the Pelger–Huett-like anomaly in the peripheral blood, which mimics features of the clonal and often pre-leukemic myelodysplastic syndromes and can cause a diagnostic dilemma in patients with a history of gastric surgery [78,80].

Copper deficiency can produce a clinical picture of anemia with polyneuropathy and myelopathy resembling subacute combined degeneration of the spinal cord due to cobalamin (vitamin B12) deficiency [83,84]. Infants and young children can also present with skeletal abnormalities resembling osteoporosis, splaying of the anterior ribs with spontaneous rib fractures, cupping and flaring of metaphyses of long bones with the formation of spurs and fractures of the submetaphysis with epiphyseal separation, which can sometimes be mistaken for signs of scurvy. Interestingly, iatrogenic copper deficiency can be produced by chronic ingestion of massive quantities of zinc, especially with zinc-containing dental fixatives, leading to microcytic anemia [85,86]. Excessive quantities of dietary zinc can impair copper absorption, leading to copper deficiency [86,87,88].

Copper deficiency can be diagnosed by decreased levels of serum ceruloplasmin or serum or 24 h urinary copper. However, ceruloplasmin acts an acute-phase reactant and is therefore less reliable than serum levels of copper [78]. Also, reference values for infants are not well defined and are usually lower than the levels observed later in life. Regardless of these restrictions, copper deficiency should be considered in patients when the serum copper level is less than 70 mcg/dL (11 μmol/L) or the ceruloplasmin level is less than 15 mg/dL after the age of 1 or 2 months. Because copper is transported by ceruloplasmin, α2-globulin, in hypoproteinemic states like exudative enteropathies and nephrosis, as well as in Wilson disease, there are low serum levels of copper. In these settings, to confirm the diagnosis of copper deficiency, an analysis of the liver copper content or clinical response after a therapeutic trial of copper supplementation can be important.

There is a prompt response of anemia and neutropenia to the administration of copper. The recommended dosage of copper in copper-deficient infants is approximately 2.5 mg of copper (~80 mcg/kg per day) oral supplementation as a copper sulfate solution [89]. IV bolus injection of copper chloride also has been used [80]. Over a 4- to 12-week period after copper supplementation, the hematological manifestations are completely reversed [90].

### 3.2. Zinc

Zinc is an essential trace element required for growth and functioning of the immune system and is a cofactor for a large number of zinc metalloenzymes, zinc-activated enzymes, and “zinc finger” transcription factors. As demonstrated by Kim et al., zinc plays an important role in erythropoiesis in the bone marrow [91]. Anemias related to quantitative hemoglobinopathies like thalassemia [92] and qualitative hemoglobinopathies like sickle cell anemia [93] may be associated with metalloprotein deficiencies. In sickle cell patients, there is decreased renal reabsorption of zinc, leading to zinc deficiency with or without associated copper deficiency [94]. Also, there was a report showing that zinc deficiency can occur in patients receiving intensive desferrioxamine therapy [95]. Zinc deficiency can also result from a loss of brush border epithelium in the small bowel as occurs in celiac disease due to the impairment of absorption, and thus zinc deficiency can also occur in combination with deficiencies of iron, folate, and vitamin B12 [96]. Animal models, cross-sectional studies in human subjects, and genetic studies demonstrate an association of the whole-body zinc status with iron homeostasis. Zinc deficiency can induce iron deficiency via a restriction of intestinal iron absorption or blocking the mobilization of iron from tissues. In vitro studies have demonstrated a role of zinc in moderating transcellular iron transport via the induction of divalent metal iron transporter-1 (DMT1) and ferroportin (FPN1) expression [97]. Therefore, a causal role of underlying zinc deficiency in the development of anemia or iron deficiency should be considered. Although zinc deficiency may lead to growth retardation, hypogonadism, impaired wound healing, altered taste sensation, immunologic abnormalities, and acrodermatitis enteropathica, there are no studies so far that indicate that isolated zinc deficiency causes anemia.

### 3.3. Selenium

Selenium deficiency typically occurs in patients living in geographic areas where the soil selenium content is very low [98]. It can also be associated with patients receiving total parenteral nutrition formulations that do not contain selenium [99,100]. A decrease in selenium is associated with a reduced level of red cell glutathione peroxidase; however, there are no reported adverse hematologic consequences. Studies have shown that low serum selenium was independently associated with anemia among older men and women in the United States [101]. Selenium is thought to protect red blood cells from hemolysis by mitigating reactive oxygen species (ROS)-mediated oxidative stress, as described by Kaur et al. [102]. Similar findings have been reported in adolescent girls living in rural Vietnam [103]. Studies have reported that in sickle cell disease patients, selenium deficiency was found to be the determining factor of hemolysis, as evaluated by the reticulocyte count, hemoglobin, indirect and total bilirubin, and lactate dehydrogenase. This suggests that nutritional supplementation with dietary selenium can reduce the risk of hemolysis in sickle cell patients [104].

## 4. Conclusions

It is apparent from this review that although iron, folate and vitamin B12 deficiencies are leading causes of anemia, deficiencies of micronutrients like vitamins other than vitamin B12 and folate, as well as trace elements like selenium, zinc and copper, can, at times, cause anemia in humans, although some of the evidence is derived from association studies and does not provide mechanistic data to support a causal relationship. Anemias due to micronutrient deficiency are poorly defined in humans, and deficiencies as seen in animal models are not always reflected in their human counterparts. These deficiencies may also be hard to identify, as most of them coexist. Manifestations may overlap and are summarized in Table 1. Fortification of food, preventing malabsorption or malnutrition and treating parasitic infestations, especially in low-income countries, could help prevent anemia due to micronutrient deficiencies.

## Figures and Tables

**Table 1:** Anemia due to unusual nutrients.

Vitamin/Trace Element	Signs and Symptoms of Deficiency	Hematologic Consequences	Monitoring	Proposed Mechanism of Anemia
Vitamin A	Night blindness, xerophthalmia	Microcytic and hypochromic anemia with anisocytosis and poikilocytosis	Serum levels of vitamin A	Impairment of iron absorption and utilization. Also plays a role in growth and differentiation of erythroid precursors
Vitamin B6 (Pyridoxine)	Dermatitis, neurological disorders, convulsions	Microcytic hypochromic anemia, ring sideroblasts	Serum levels of vitamin B6	Impairment of hemoglobin synthesis via the porphyrin synthetic pathway where pyridoxine acts as a cofactor
Pantothenic acid	Peripheral neuropathy	Anemia	NA	Mechanism of action unclear
Niacin (Vitamin B3)	Pellagra (dementia, diarhea and dermatitis.“3 D’s”))	Anemia	Serum levels of vitamin B3	Mechanism of action unclear
Riboflavin (Vitamin B2)	Fatigue, dermatitis, neurological dysfunction	Vacuolated red cell precursors and red cell aplasia	Serum levels of vitamin B2	Impaired iron release from ferritin Induction of glutathione reductase deficiency
Thiamine (Vitamin B1)	Beriberi (Dry and Wet), Wernicke and Korsakoff syndromes, Rogers syndrome	Megaloblastic anemia	Serum levels of vitamin B1	Induces cell-cycle arrest or apoptosis in marrow cells
Vitamin C	Scurvy, capillary hemorrhages, infection, bleeding	Megaloblastic anemia	Serum levels of vitamin C	Failure to synthesize tetrahydrofolate or protect it from oxidation ultimately results in megaloblastic anemia Compromised intestinal iron absorption due to failure of reduction of ferric to more soluble ferrous state
Vitamin E	Visual problems, neuropathies and myopathies	Hemolytic anemia, thrombocytosis	Serum levels of vitamin E	Anemia often is associated with fragmentation and other morphologic alterations of the erythrocytes as alpha tocopherol is an antioxidant preventing oxidant damage to red cell membrane
Copper	Neurological abnormalities, skeletal abnormalities, depigmented hair, cardiac defects	Ring sideroblasts, macrocytic, normocytic and less commonly microcytic anemia, vacuolated erythroid and granulocytic precursors, neutropenia	Serum ceruloplasmin Serum or urinary 24 h copper	Hinders absorption and utilization of iron
Zinc	Growth retardation, acrodermatitis enetropathica, impaired taste sensation and delayed wound healing	Microcytic anemia due to concomitant iron deficiency	Serum zinc levels	Impairs iron absorption
Selenium	Cardiomyopathy, muscular myopathy	Macrocytic anemia or hemolytic anemia	Plasma/serum selenium	Mechanism of anemia unclear

## Data Availability

No new data were created or analyzed in this study.
